# Identification of a prognostic immune‐related signature for small cell lung cancer

**DOI:** 10.1002/cam4.4402

**Published:** 2021-11-05

**Authors:** Qi Xie, Huili Chu, Jian Yi, Hui Yu, Tiantian Gu, Yaping Guan, Xiaolin Liu, Jing Liang, Yan Li, Jun Wang

**Affiliations:** ^1^ Department of Oncology The First Affiliated Hospital of Shandong First Medical University & Shandong Provincial Qianfoshan Hospital Shandong Key Laboratory of Rheumatic Disease and Translational Medicine Shandong Lung Cancer Institute Jinan China; ^2^ Department of Oncology No. 960 Hospital The People's Liberation Army of China Jinan China; ^3^ YuceBio Technology Co., Ltd. Shenzhen China

**Keywords:** immune cell infiltration, immune‐related gene, prognosis, signature, small cell lung cancer, tumor mutational burden

## Abstract

**Purpose:**

As a subgroup of lung cancer, small cell lung cancer (SCLC) is characterized by a short tumor doubling time, high rates of early occurred distant cancer spread, and poor outcomes. Despite its exquisite sensitivity to chemotherapy and radiotherapy, acquired drug resistance and tumor progression are typical. This study aimed to develop a robust signature based on immune‐related genes to predict the outcome of patients with SCLC.

**Methods:**

The expression data of 77 SCLC patients from George's cohort were divided into training set and testing set, and 1534 immune‐related genes from ImmPort database were used to generate and validate the signature. Cox proportional hazards and the Kaplan–Meier analysis were used for developing and testing the prognostic signature. Single‐sample gene set enrichment analysis was used to determine immune cell infiltration phenotypes.

**Results:**

A 10‐gene model comprising *NR3C1*, *NR1D2*, *TANK*, *ARAF*, *HDGF*, *INHBE*, *LRSAM1*, *PLXNA1*, *PML*, and *SP1* with the highest frequency after 1000 interactions, was chosen to construct immune‐related signature. This signature showed robust predictive value for SCLC patients’ survival in both training and testing sets. This signature was weakly associated with the clinic pathological values like TNM stage. Furthermore, patients with low risk presented with activation of immune signal pathways, and specific immune cell infiltration with high levels of CD56^bright^ NK cells but low levels of CD8^+^ T cells, mast cells, and helper T cells.

**Conclusion:**

The present study developed immune‐related signature that may help predict the prognosis of SCLC patients, which reflects an unappreciated level of heterogeneity of immunophenotype associated with diverse prognosis for specific subsets in this highly lethal cancer type.

## Graphical abstract

Here, we constructed a prognostic immune‐related signature for predicting small cell lung cancer (SCLC) patients’ survival. The 10‐gene signature was enriched in growth factor activity and immune‐related TGF‐β signaling pathway. Furthermore, increased CD56dim NK cells and reduced plasmacytoid dendritic cells infiltration were significantly associated with survival prolongment. However, SCLC patients with low risk presents more CD56bright NK cells but less CD8^+^ T cells, mast cells, and helper T cells infiltration compared to those with high risk. Our findings indicate that this immune‐related signature may help predict the prognosis of SCLC patients, which reflects an unappreciated level of heterogeneity of immunophenotype associated with diverse prognosis for specific subsets in this highly lethal cancer type.

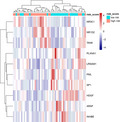

## 1 INTRODUCTION

Lung cancer, the most common cancer in male and female worldwide, accounts for approximately 19% of all cancer deaths.^1^ In general, a majority of lung cancers are non‐small cell lung cancer (NSCLC), whereas 13%–15% is small cell lung cancer (SCLC). SCLC is an aggressive undifferentiated neuroendocrine tumor and clinically characterized by its high grade, rapid growth, and early spread of cancer cells. Thus, approximately 70% of SCLC patients are classified as having extensive disease, which leads to the extremely poor prognosis.^2^ Although first‐line chemotherapy with etoposide plus either cisplatin or carboplatin produces a high response rate of up to 70%, SCLC patients fail to have an opportunity to receive molecular‐targeted therapy targeting specific driver genes. Furthermore, most patients relapse within 6 months of the completion of initial treatment due to acquired drug resistance, subsequent effective treatment options are still limited.^3^ SCLC has been reported to have high tumor mutation burden and high neoantigens formation which are associated with increased sensitivity to immunotherapy with immune checkpoint inhibitors (ICIs).^4^ Actually, ICIs targeting the programmed cell death 1 and programmed cell death‐ligand 1 (PD‐L1) pathway, such as nivolumab, pembrolizumab, atezolizumab, and durvalumab monotherapy or in combination with chemotherapy have been shown to prolong the survival of patients with SCLC with manageable toxicity profile.^3^ However, the application of ICIs in SCLC appears to be less effective when compared to NSCLC, and only a minority of SCLC patients can benefit from immune checkpoint blockade.^4^ In particular, low expression levels of major histocompatibility complex and PD‐L1 on tumor cells, less immune cells infiltration, and high ratio of suppressive immune cells all have compromised the efficacy of ICIs.

The importance of tumor immune microenvironment in SCLC has been demonstrated using antigen vaccines and dendritic cell vaccines treatment.^5^, ^6^ However, there is a lack of feasible cytogenetic signatures associated with immune microenvironment to predict SCLC patients' prognosis.^2^, ^3^ Therefore, it is essential to define immune‐related biomarkers as a predictor for SCLC patients' survival from the perspective of tumor immunity, which could help clinician identify a subgroup with a favorable outcome and might benefit from immunotherapy with ICIs. In this study, transcriptome data were utilized to create an immune‐related signature comprising 10 genes for SCLC prognostication.

## 2 MATERIALS AND METHODS

### 2.1 Construction of the immune‐related risk signature

Here we constructed a prognostic signature by focusing on immune‐related genes, which were downloaded from the ImmPort database (https://immport.niaid.nih.gov). ImmPort database is one of the largest open repositories of human immunological data.^7^ We downloaded a list of 2,498 immune‐related genes from ImmPort database (Table S1). A variety of immune‐related genes were included, such as cytokine genes, cytokine receptor genes, and genes associated with the T‐cell receptor signaling pathway, B‐cell antigen receptor signaling pathway, natural killer cell cytotoxicity, antigen processing, and presentation pathways. All patients from George's cohort^7^ were obtained, and 77 samples with OS information were randomly divided into a training set (*n* = 54) for identifying key immune‐related genes and a testing set (*n* = 23) for validating the immune‐related genes signature. The clinical and survival information of the 77 samples are summarized in Table 1. Univariate analysis was performed to identify prognostic immune‐related risk signature, and *p* < 0.05 indicates a significant correlation between immune‐related genes and prognosis. In order to identify the best gene model for predicting the outcome in SCLC patients, the Cox proportional hazards model with an elastic net penalty (iteration = 1000) was performed with R3.4.4 package “glmnet.” The penalty parameter was evaluated by 10‐fold cross‐validation with the training dataset. Based on a linear combination of Cox coefficient and gene expression, genes weighted value was yielded for further analysis.

**TABLE 1:** Clinical characteristics of the total datasets

Feature	Sample number	Ratio (%)
Age		
≤60 years	20	26.0
>60 years	57	74.0
Gender		
Male	54	70.1
Female	23	29.9
AJCC stage		
Stage I	33	42.9
Stage II	14	18.2
Stage III	21	27.3
Stage IV	9	11.7

### 2.2 Performance assessment

The predictive efficiency of the immune‐related risk signature was assessed using Harrell's concordance index (C‐index) and time‐dependent receiver operating characteristic (ROC) analysis. The area under curve (AUC) was calculated using the “survival ROC” package in R3.4.4. In order to estimate survival differences of patients between high‐ and low‐risk groups, the Kaplan–Meier (K–M) survival curves were generated using the “survminer” package in R. Besides, principal component analysis (PCA) was performed to assess gene expression patterns.

### 2.3 Gene enrichment analysis

In order to explore the biological processes of differentially expressed genes (DEGs), Gene Ontology (GO) and Kyoto Encyclopedia of Genes and Genomes (KEGG) pathway analyses were performed with the Database for Annotation, Visualization, and Integrated Discovery (https://david.ncifcrf.gov/) with the cut‐off criterion of false discovery rate < 0.01. *p* < 0.05 was considered statistically significant. The 26 immune cell types enrichment score was calculated using single‐sample gene set enrichment analysis (ssGSEA) method implemented by R package Gene Set Variation Analysis (GSVA), to measure the level of immune cell infiltration.^8^, ^9^

### 2.4 Statistical analysis

Heatmaps were produced using R pheatmap package. Clustering of the heatmaps was performed by the standard R hclust (hierarchical clustering) method, using the “ward.D2” option. Multivariable cox analysis was performed with cox proportional hazard regression using R3.4.4 survival package for three datasets: (1) risk score, age, gender, and pathological stage; (2) proportion of eight immune cells infiltrated; and (3) 26 immune cells enrichment score. We obtained the gene set corresponding to the 26 immune cells mentioned in previous research and used the default parameters of the ssGSEA algorithm for immune cell infiltration analysis.^10^ The boxplots were conducted using the R package called ggpubr. Differences among two and three groups were determined by the Wilcoxon rank‐sum test and the Kruskal–Wallis test, respectively. *p* < 0.05 was considered statistically significant.

## 3 RESULTS

### 3.1 Construction and validation of the immune‐related risk signature

**FIGURE 1:**
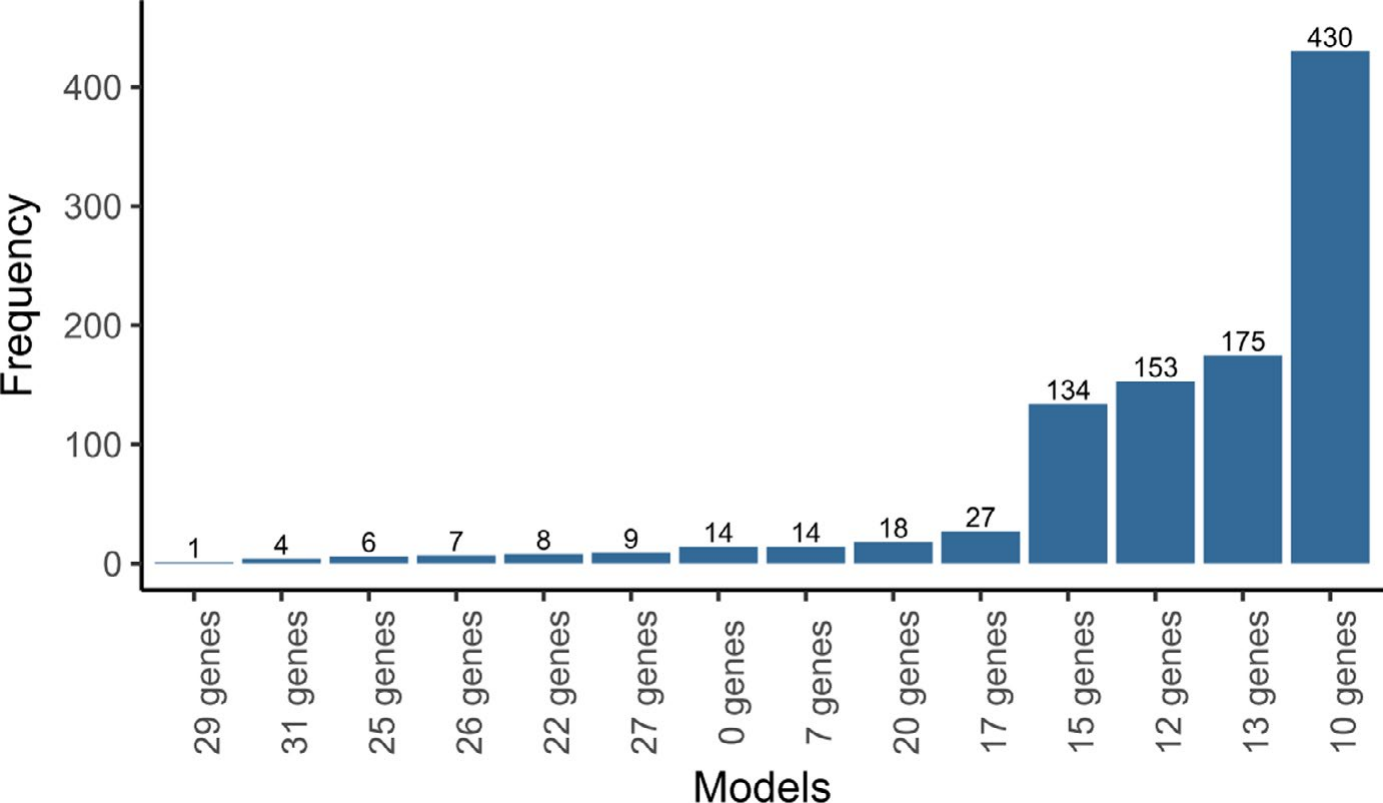
Frequency of each model in 1000 iterations. Generation of 14 model feature gene sets after 1000 iterations. One gene model contained 10 feature genes was highly stable and reaches the frequency of 430 times compared with other 13 gene models

All 77 samples were randomly divided into a training set (*n* = 54) (54/77, 70% for identifying key genes) and a testing set (*n* = 23) (23/77, 30% for validating). Using univariate Cox analysis, the correlation between gene expression and patient's overall survival (OS) was calculated and 77 genes with prognostic ability were obtained (*p* < 0.05). In order to develop the best gene model to predict the prognosis of SCLC patients, the Cox proportional hazards model with an elastic net penalty was performed. After 1000 iterations, 14 model feature gene sets were obtained, and one of which contained 10 feature genes was highly stable and reaches the frequency of 430 times, accounting for 43% in 1000 iterations (Figure 1). This 10‐gene model include *ARAF*, *HDGF*, *INHBE*, *LRSAM1*, *NR1D2*, *NR3C1*, *PLXNA1*, *PML*, *SP1*, and *TANK* and respective coefficients are listed in Table 2. Using the risk scoring formula as follows, the risk score for each SCLC patient was calculated based on expression level and coefficient of 10 characteristic genes.

$$\begin{array}{cc} \text{Risk}\;\text{score} & =(‐0.0066322*\text{ARAF})+(‐0.0015719*\text{HDGF})+(‐0.0021426*\text{INHBE})\\ & \;+(‐0.0152107*\text{RSAM}1)+(0.00920882*\text{NR}1D2)+(0.0185948*\text{NR}3C1)\\ & \;+(‐0.0009105*\text{PLXNA}1)+(‐0.0081578*\text{PML})+(‐0.0023929*\text{SP}1)+(0.00671622*\text{TANK}). \end{array}$$

**TABLE 2:** The best gene set and coefficient related to prognosis

Gene	Coef
ARAF	−0.0066322
HDGF	−0.0015719
INHBE	−0.0021426
LRSAM1	−0.0152107
NR1D2	0.00920882
NR3C1	0.0185948
PLXNA1	−0.0009105
PML	−0.0081578
SP1	−0.0023929
TANK	0.00671622

**FIGURE 2:**
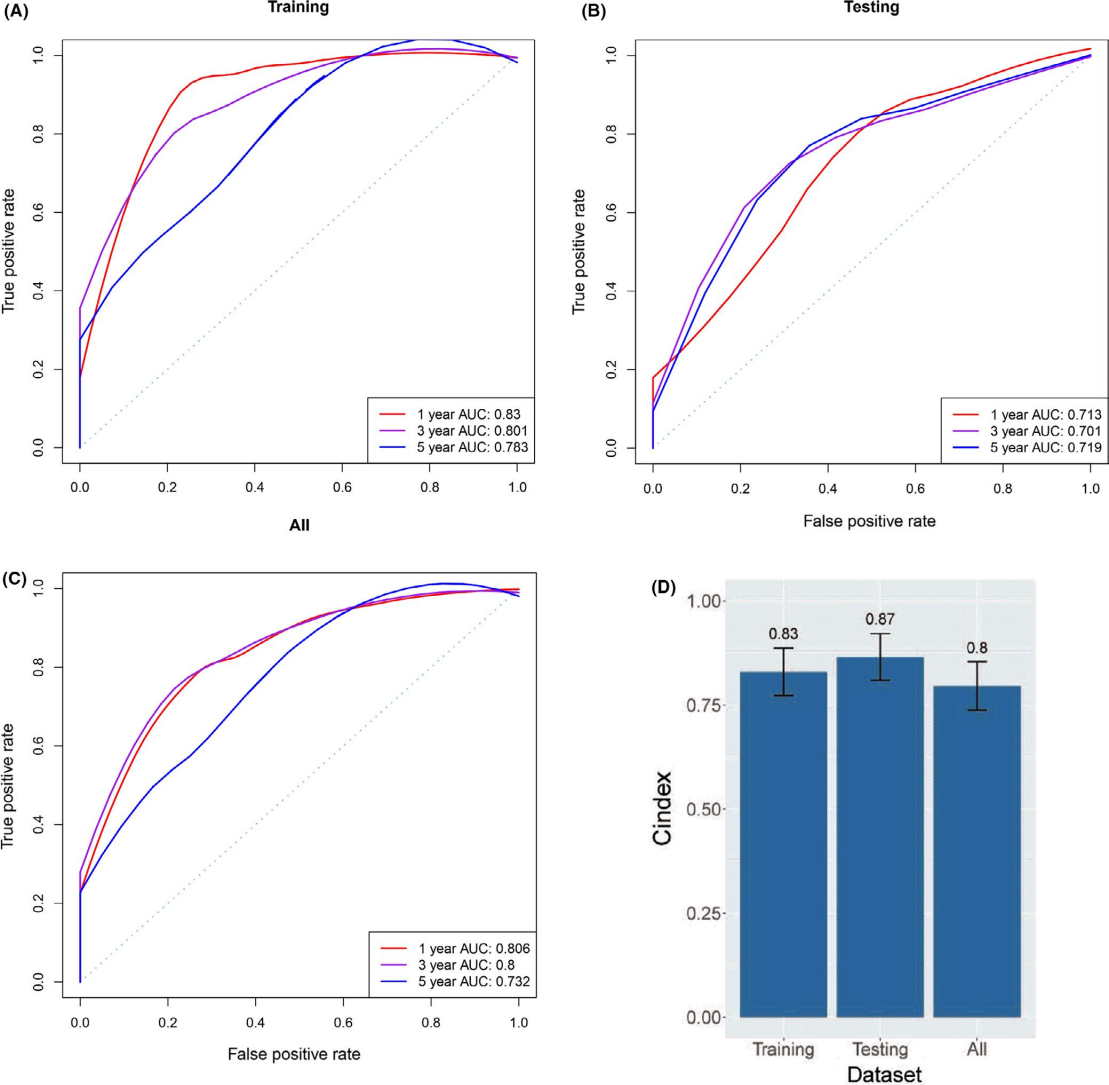
Model performance evaluation. Receiver operator characteristic analysis was performed to compare our 10‐gene signature in predicting 1‐, 3‐, and 5‐year overall survival in training (A), testing (B), and all data cohorts (C). Harrell's concordance index (C‐index) for the training, testing, and total data set was 0.83, 0.87, and 0.8, respectively (D). AUC, area under curve

Time‐dependent ROC and C‐index were applied to evaluate the prognostic values of the 10‐gene signature in terms of OS. The ROC curve analysis of 10‐gene signature in the training set has exhibited the favorable predictive value for survival of SCLC patients, and AUC was 0.83 at 1 year, 0.801 at 3 year, and 0.783 at 5 year (Figure 2A). Then, 10‐gene signature was validated in the testing set, and the 1‐, 3‐, and 5‐year AUC were 0.713, 0.701, and 0.719, respectively (Figure 2B). As for all cohorts, 10‐gene signature also achieved an accuracy to predict patient's OS, and the AUC value for 1‐, 3‐ and 5‐year was 0.806, 0.8, and 0.732, respectively (Figure 2C). Besides, the C‐index for the training, testing, and total data set was all above 0.75 (Figure 2D), indicating a superior prognostic value of constructed model.

### 3.2 Association between 10 immune‐related risk signature and SCLC patients' survival

**FIGURE 3:**
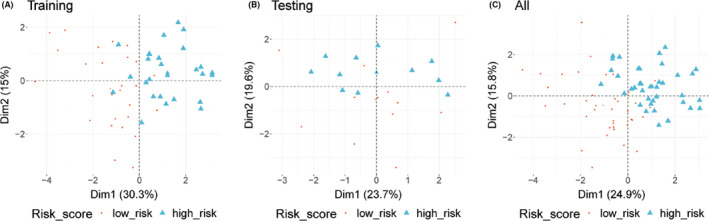
Principal component analysis analysis. Distribution patterns for high‐ and low‐risk population based on 10 genes. Most of the high‐risk population are well separated from the low‐risk population, displaying a different distribution pattern of high‐risk and low‐risk population. (A) Training data set. (B) Testing data set. (C) All data set

**FIGURE 4:**
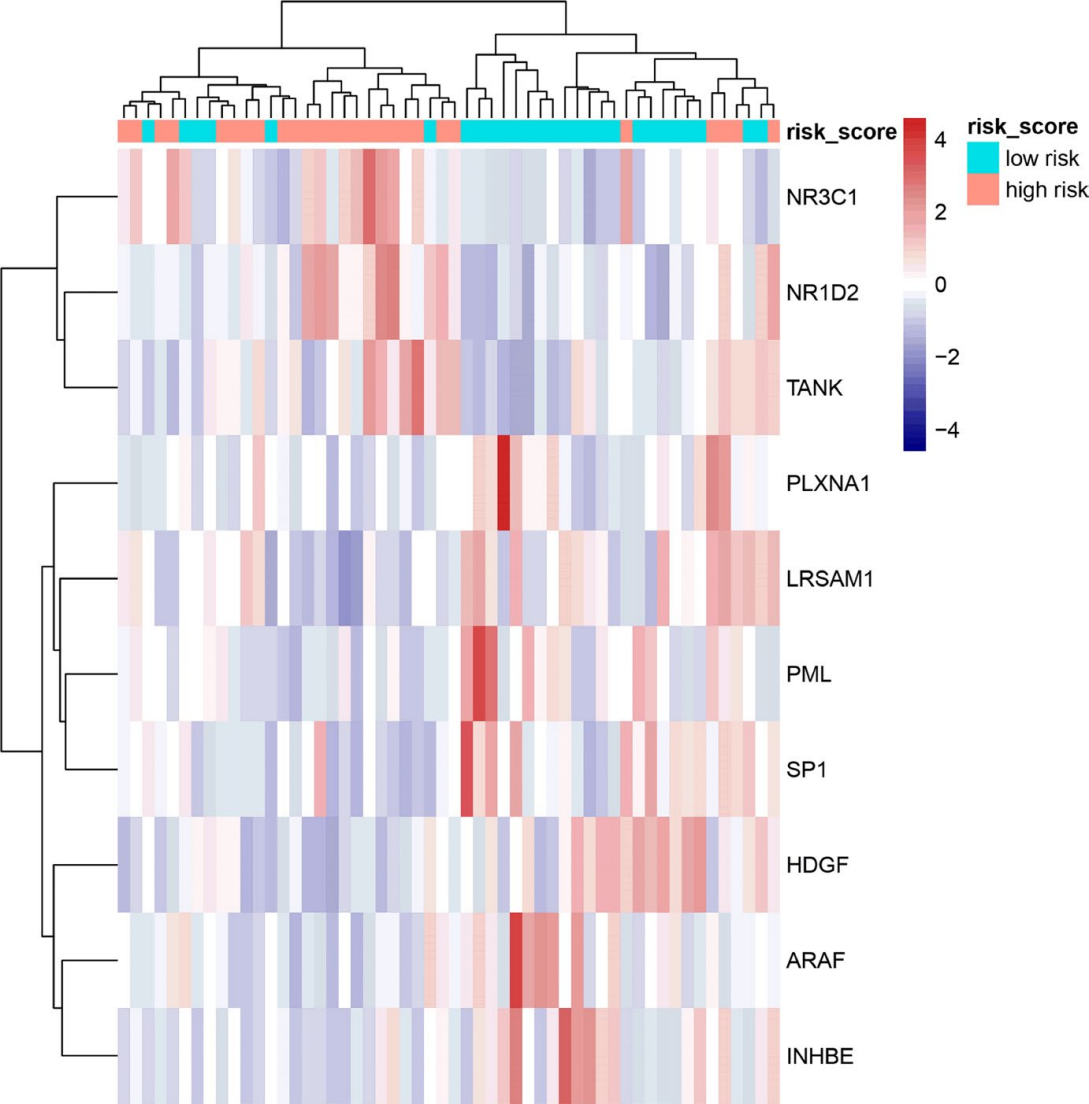
Cluster analysis of 10 characteristic genes expression in training data set. Ten genes with higher expression in the heatmap are shown in red color, and with lower expression are shown in blue. Tiffany blue represents cancer tissue from the low‐risk population, while the pink represents cancer tissue from the high‐risk population

The median value of the risk score is taken as the threshold to divide the high‐risk and low‐risk populations. PCA of the training, testing, and total SCLC cohort demonstrated a different distribution pattern of high risk and low risk based on 10 immune‐related gene expression, indicating their difference in immune phenotype (Figure 3), the training set was clustered and heatmap was created (Figure 4), and the *NR3C1*, *NR1D2*, and *TANK* gene expression levels were higher in high‐risk population, while *ARAF*, *HDGF*, *INHBE*, *LRSAM1*, *PLXNA1*, *PML*, and *SP1* gene expression levels were higher in low‐risk population (Figure 4).

**FIGURE 5:**
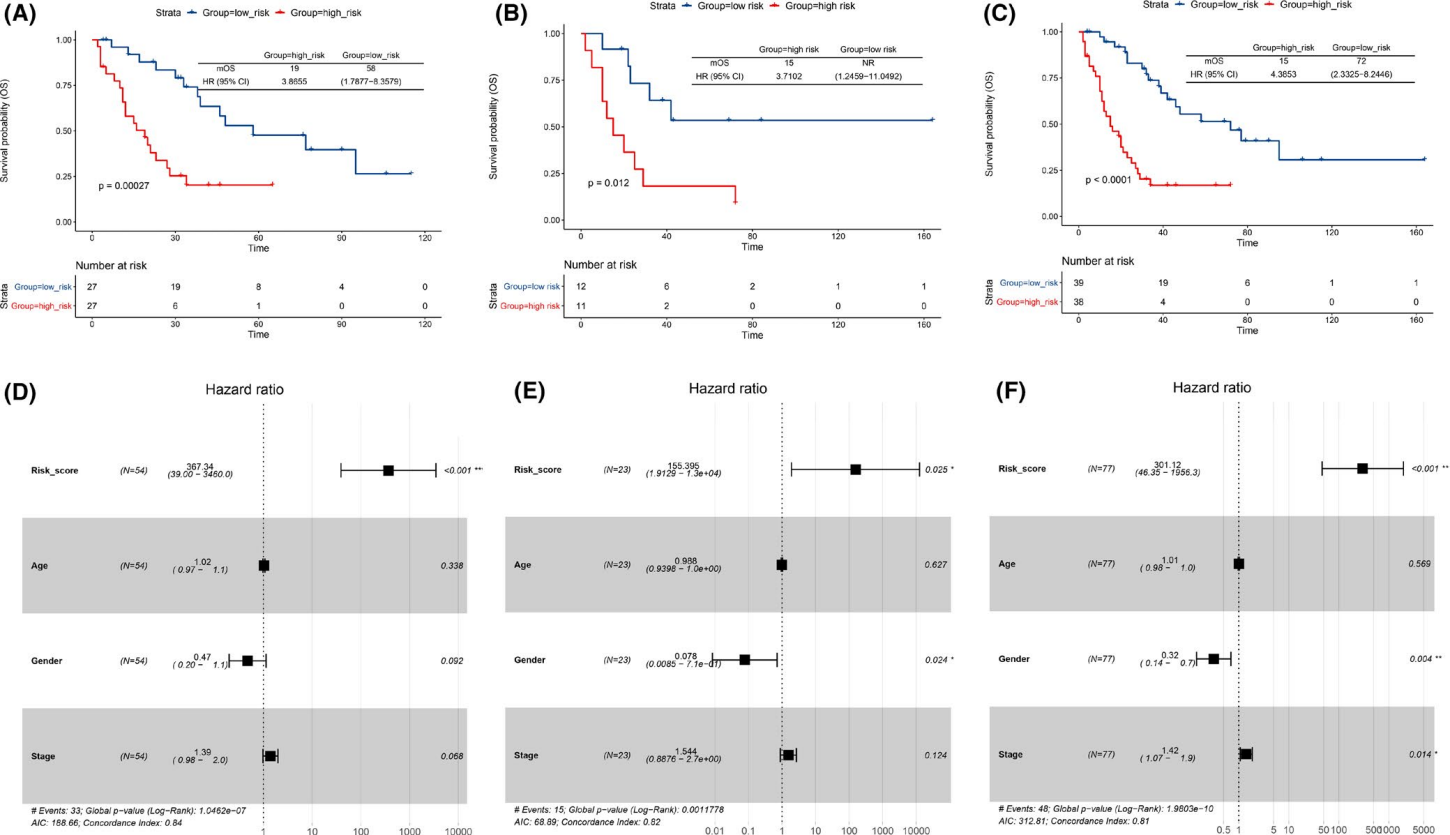
K–M survival and hazard ratio analysis. The Kaplan–Meier curves of overall survival (OS) for SCLC patients with high risk and low risk in training set (A), testing set (B), and all data set (C). Hazard ratios (HRs) and 95% CIs are for high‐risk group versus low‐risk population. *p* values were calculated with the log‐rank test. Gender (*p* = 0.024) in testing set (E) and gender (*p* = 0.004) and stage (*p* = 0.014) in all data set (*p* = 0.004) (F) were significantly related to the prognosis by Cox regression analysis

In order to calculate the association between immune‐related risk signature and SCLC patients' survival outcome, the K–M survival analysis was performed in three data sets. In the training sets, SCLC patients from the low‐risk group had significantly better OS than patients from the high‐risk group (HR = 3.87, 95% CI: 1.79–8.36, *p* = 0.00027) (Figure 5A). The same trends were also observed in the validation sets (HR = 3.71, 95% CI: 1.25–11.05, *p* = 0.012) (Figure 5B) and total data sets (HR = 4.39, 95% CI: 2.33–8.24, *p* < 0.0001) (Figure 5C). Hazard ratio analysis showed risk score was a poor prognostic factor of the risk of survival in SCLC patients with a HR of 367.34 in training set (95% CI: 39–3460, *p* < 0.001), and 155.40 in testing set (95% CI: 1.91–13000, *p* = 0.025). (Figure 5D–F). In addition, in the validation and total data set, the gender of SCLC patients was a favorable prognostic factor of the risk of survival, and the risk of survival was significantly lower in female SCLC patients (HR = 0.078, 95% CI: 0.0085–0.71, *p* = 0.024; HR = 0.32, 95% CI: 0.14–0.70, *p* = 0.004) (Figure 5D–F). In the total data set, pathological stage is a poor prognostic factor of the risk of survival in SCLC patients (HR = 1.42, 95% CI: 1.07–1.9, *p* = 0.014). Of note, there was no significant association between the age and survival risk of SCLC patients in all three data sets (Figure 5D–F).

### 3.3 Enrichment of GO and KEGG pathway by immune‐related risk signature

To elucidate the molecular mechanism of the 10 immune‐related risk gene signature, GO and KEGG pathway enrichment analyses were applied to explore the functions of the 10 genes. Fifteen Go terms were significantly enriched in GO enrichment and four pathways were enriched in KEGG pathway enrichment analyses. KEGG pathway enrichment analyses revealed that the DEGs participated in acute myeloid leukemia (*p* = 0.04), TGF‐β signaling pathway (*p* = 0.04), endocrine resistance (*p* = 0.04), and parathyroid hormone synthesis, secretion, and action (*p* = 0.04) (Table 3). After GO enrichment analyses, the 10 genes were significantly enriched in biological processes including small ubiquitin‐like modifier binding, core promoter binding, transcription factor activity, RNA polymerase II transcription factor binding, steroid hormone receptor activity, ubiquitin‐like protein binding, core promoter sequence‐specific DNA binding, growth factor activity, and ubiquitin protein ligase binding (Table 4).

**TABLE 3:** Pathways with significant enrichment of characteristic genes

ID	Description	*q* value
hsa05221	Acute myeloid leukemia	0.04
hsa04350	TGF‐beta signaling pathway	0.04
hsa01522	Endocrine resistance	0.04
hsa04928	Parathyroid hormone synthesis, secretion, and action	0.04

**TABLE 4:** Go term with significantly enriched characteristic genes

ID	Description	*q* value	Gene ID
GO:0032183	SUMO binding	8.65E–04	NR3C1/PML
GO:0001047	Core promoter binding	9.74E–04	NR1D2/NR3C1/SP1
GO:0001076	Transcription factor activity, RNA polymerase II transcription factor binding	1.57E–03	HDGF/NR1D2/NR3C1
GO:0003707	Steroid hormone receptor activity	4.01E–03	NR1D2/NR3C1
GO:0032182	Ubiquitin‐like protein binding	5.80E–03	NR3C1/PML
GO:0001046	Core promoter sequence‐specific DNA binding	5.80E–03	NR1D2/SP1
GO:0000982	Transcription factor activity, RNA polymerase II proximal promoter sequence‐specific DNA binding	6.49E–03	NR1D2/NR3C1/SP1
GO:0008083	Growth factor activity	1.33E–02	HDGF/INHBE
GO:0001077	Transcriptional activator activity, RNA polymerase II proximal promoter sequence‐specific DNA binding	2.31E–02	NR3C1/SP1
GO:0031625	Ubiquitin protein ligase binding	2.43E–02	PML/TANK
GO:0044389	Ubiquitin‐like protein ligase binding	2.50E–02	PML/TANK
GO:0001228	Transcriptional activator activity, RNA polymerase II transcription regulatory region sequence‐specific DNA binding	2.70E–02	NR3C1/SP1
GO:0000978	RNA polymerase II proximal promoter sequence‐specific DNA binding	2.70E–02	NR3C1/SP1
GO:0000987	Proximal promoter sequence‐specific DNA binding	2.70E–02	NR3C1/SP1
GO:0048018	Receptor ligand activity	2.70E–02	HDGF/INHBE

### 3.4 Correlation of the immune‐related risk signature with clinicopathologic features

**FIGURE 6:**
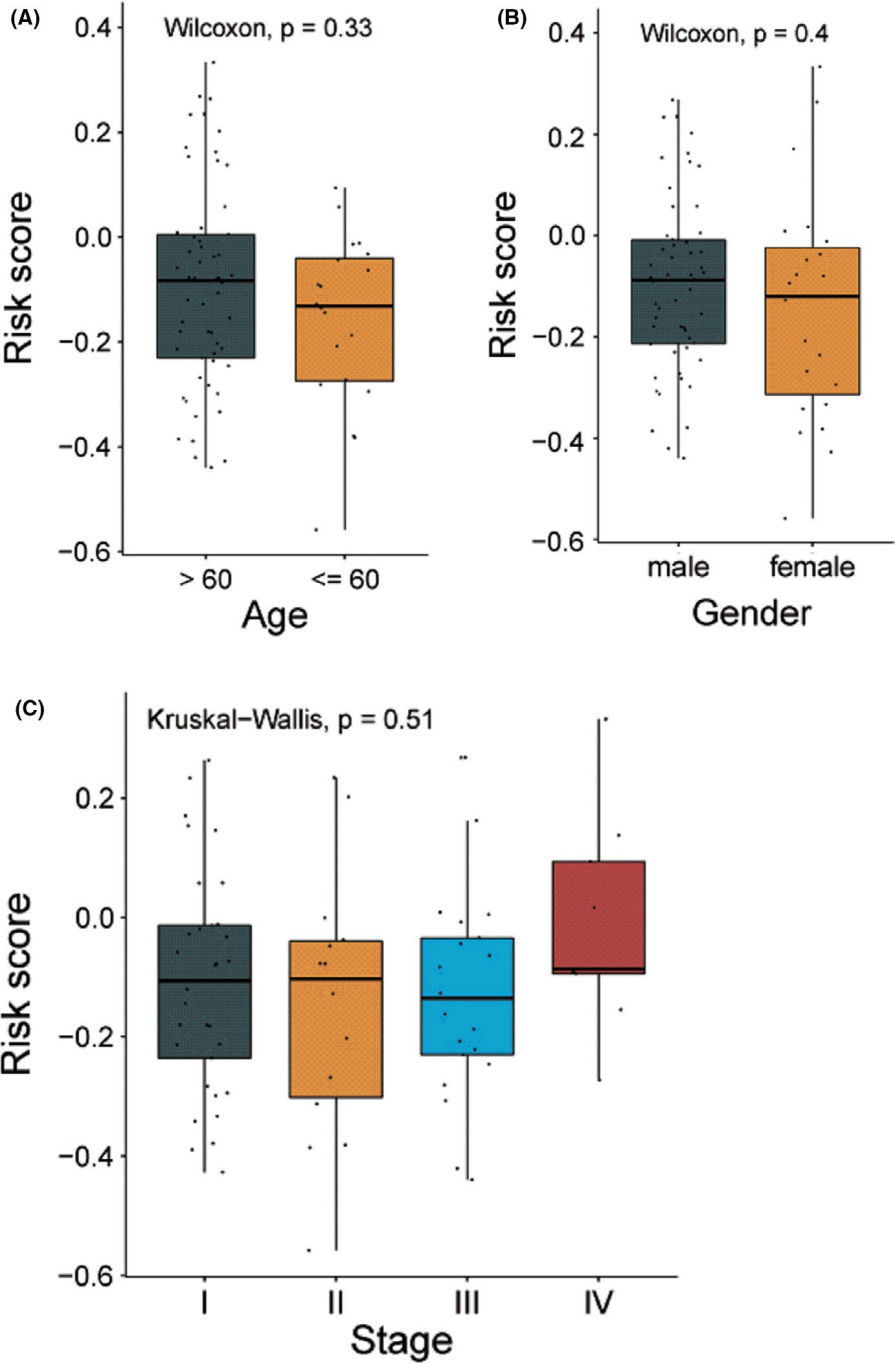
Difference test of risk score between different pathological stages. (A) Comparison of risk score between small cell lung cancer (SCLC) patients who were above 60 years old and those at or below 60 years old. (B) Comparison of risk score between male and female SCLC patients. (C) Comparison of risk score between SCLC patients at different pathological stages

The relationship between 10‐gene signature and tumor staging, age, and gender was analyzed. We have observed that SCLC patients who were above 60 years old (Figure 6A) and male SCLC patients (Figure 6B) tend to have higher risk. In our case, the numbers of stages I, II, III, and IV patients in dataset were 33, 14, 21, and 9, respectively. We have found that the mean of risk score in advanced SCLC was higher than early stage SCLC, but the difference was not significant (Figure 6C).

### 3.5 Tumor immunity relevance of immune‐related risk signature

**FIGURE 7:**
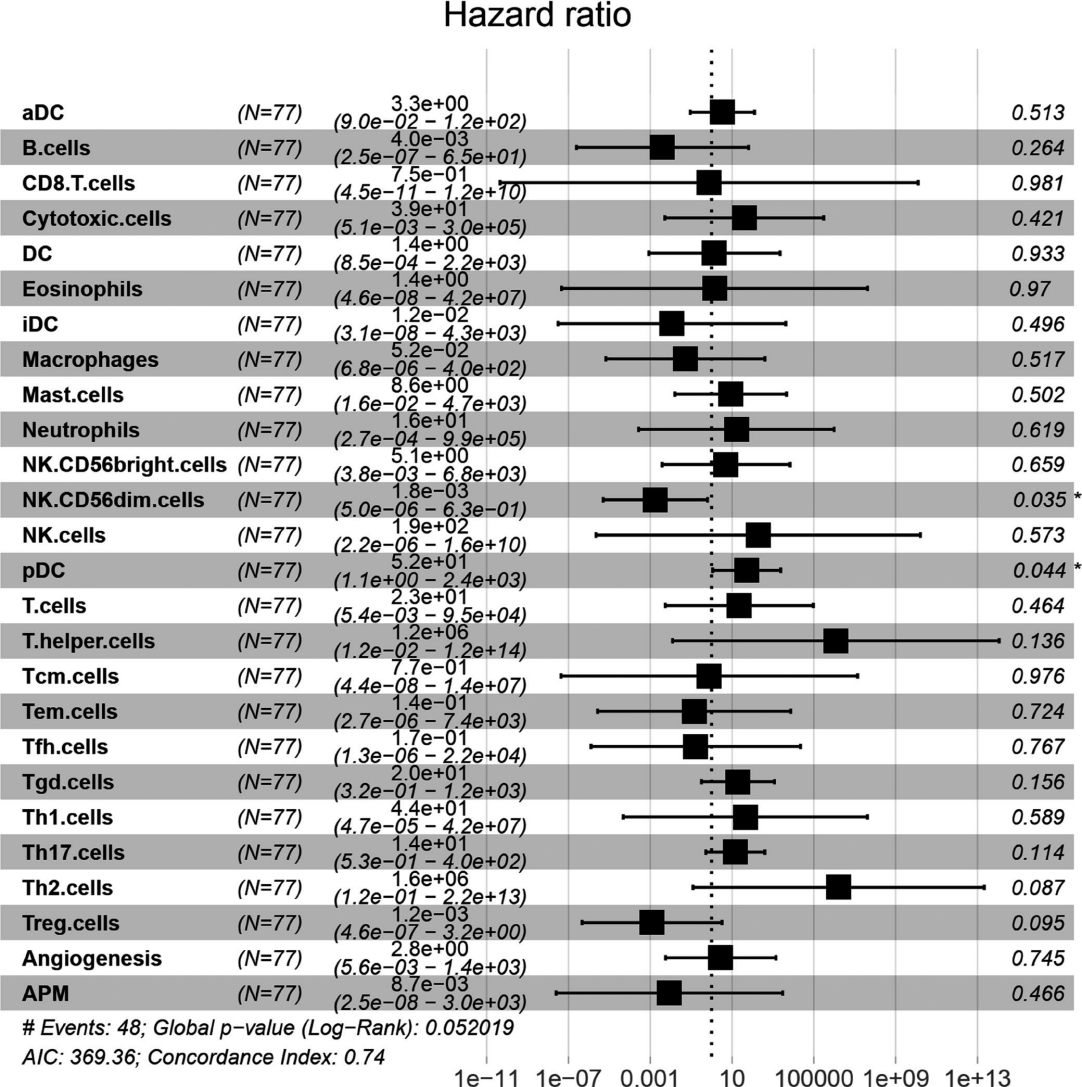
Hazard ratio analysis of score values of immune cells. The relationship between abundance of immune cells and overall survival was investigated. NK56^dim^ cells (*p* = 0.035) and pDC (*p* = 0.044) were significantly related to the prognosis in multivariate Cox regression model. APM, antigen‐presenting machinery; DC, dendritic cell; HR, hazard ratios; NK, natural killing cell; Tcm, central memory T cells; Tem, effector memory T cells; Tfh, follicular helper T cells; Treg, regulatory T cells

The abundance of 26 immune cells in the total data set was calculated by ssGSEA method. The relationship between the abundance of immune cells in tumor immune microenvironment and overall survival (OS) was analyzed by multivariate Cox analysis. We have observed that abundance of specific immune cells was associated with OS of SCLC patients. The abundance of CD56^dim^ NK cells is a favorable prognostic factor for survival of SCLC patients (*p* = 0.035), while the abundance of the plasmacytoid dendritic cells (pDC) is a poor prognostic factor for survival of SCLC patients (*p* = 0.044) (Figure 7). There was no significant association between other immune cell subsets including CD8^+^ T cells, macrophages, or T cells and increased patients' survival.

**FIGURE 8:**
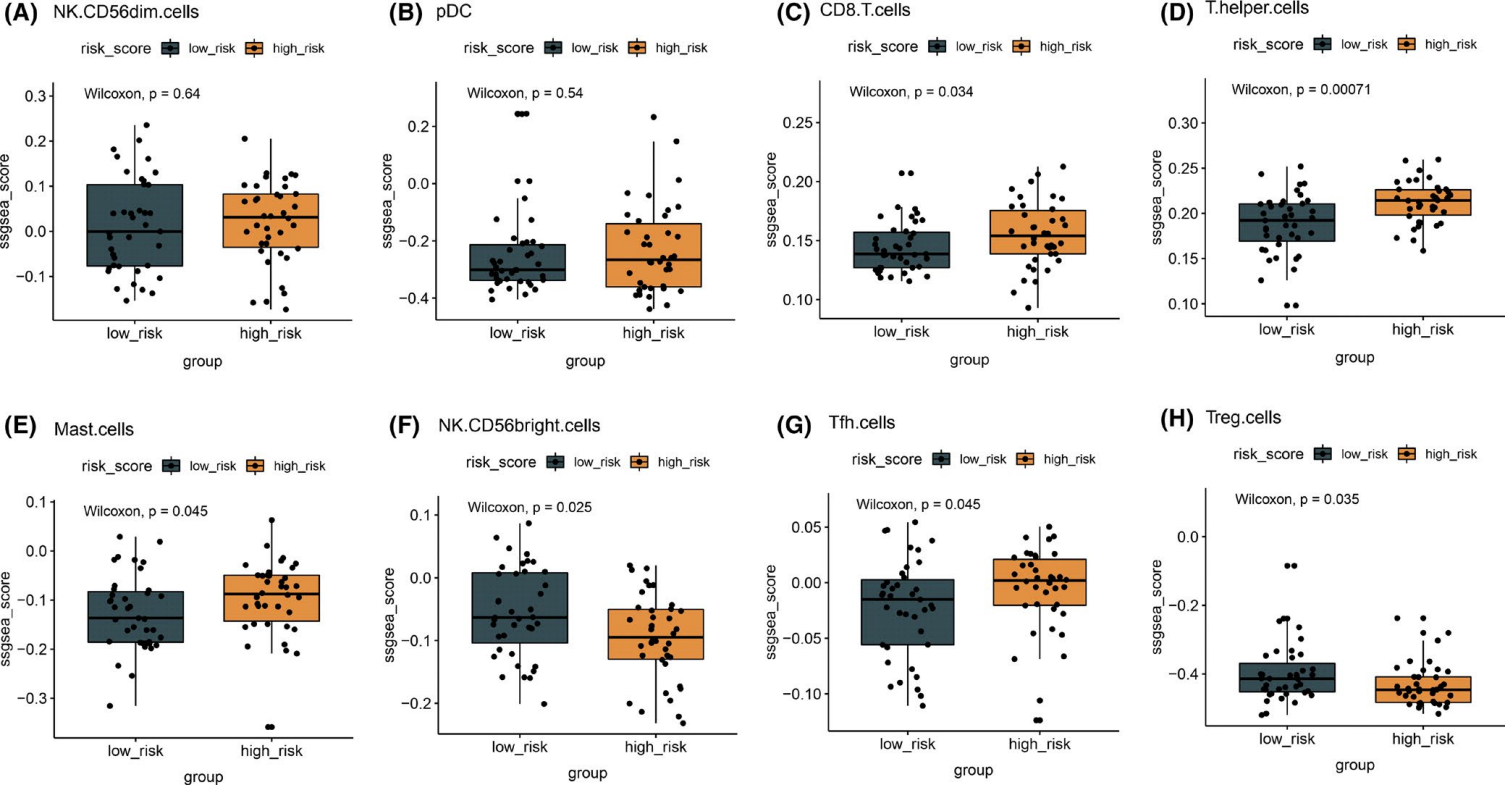
Difference in immune cell score in patients with high‐ and low‐risk score. The abundance of different immune cell infiltration status between high‐ and low‐risk populations is analyzed, and results are shown in box plots. *p* < 0.05 was considered statistically significant

In order to interpret survival difference between high‐ and low‐risk population from the perspective of tumor immunity, the immune cell infiltration profile in patients with high and low risk was analyzed. We failed to observe a significant difference regarding CD56^dim^ NK cells and pDC infiltration between low‐risk and high‐risk groups, indicating that immune‐related risk signature and immune microenvironment have independent effects on prognosis (Figure 8A,B). In addition, patients with high risk had more CD8^+^ T cells, helper T cells, mast cells, and follicular helper T (Tfh) cells but less Treg cells compared to those with low risk. Interestingly, patients with low risk had more CD56^bright^ cell infiltration than patients with high risk (Figure 8C–H).

## 4 DISCUSSION

Lung cancer is the leading cause of cancer‐related death worldwide. Based on the histological differences, lung cancer is broadly classified into two subtypes: SCLC and NSCLC. SCLC comprises about 15% of all lung cancer cases.^11^ Given that SCLC is an incurable cancer type, it is essential to develop immune‐related biomarkers to identify patients who have a good prognosis and might benefit more from immunotherapy.^12^ Here, we constructed a prognostic immune‐related signature for predicting SCLC patients' survival. The 10‐gene prognostic immune‐related signature was enriched in growth factor activity and immune‐related TGF‐β signaling pathway. Furthermore, increased CD56^dim^ NK cells and reduced pDC infiltration were significantly associated with survival prolongment. However, according to the present prognostic immune‐related signature, SCLC patients with low risk presents more CD56^bright^ NK cells but less CD8^+^ T cells, mast cells, and helper T cells infiltration compared to those with high risk. Our findings indicate that the present study developed immune‐related signature that may help predict the prognosis of SCLC patients, and SCLC has an unappreciated level of heterogeneity of SCLC immunophenotype that determines the diverse prognosis for specific subsets.

The immune‐related signature consisted of 10 immune‐related genes with prognostic ability. Three (NR3C1, Sp1, and PML) of the genes in the 10‐gene signature were previously reported to be associated with SCLC. ***NR3C1*** (nuclear receptor subfamily three group C member 1) gene encodes glucocorticoid receptor (GR). GR displays anti‐inflammatory effects through transcriptional activation of glucocorticoid‐induced leucine zipper genes^13^ or transrepression via interferences with the activity of many other immune‐related transcription factors, including nuclear factor‐κB, nuclear factor of activated T cells, activator protein 1, interferon regulatory factor 3, cyclic‐AMP response binding protein, T‐box transcription factor 21, GATA binding protein 3,^14^ and higher NR3C1 expression in high‐risk group patients who have worse OS might compromise pro‐inflammatory and antitumor immune response in vivo. ***Sp1*** gene encoded Sp1 protein which is a well‐known zinc finger transcription factor.^15^ Zhu et al. have reported Sp1 directly regulate decoy receptor 3 (DcR3) expression in hepatocellular carcinoma which promotes Th2 and Treg cell differentiation but inhibits Th1 cell differentiation.^16^ DcR3 expression was also significantly higher in SCLC cancer tissues compared to normal lung tissue,^17^ thus inhibition of DcR3 expression by interfering with upstream Sp1 pathway may provide a novel immunotherapeutic target to restore antitumor immune response in low‐risk group SCLC patients. ***PML*** (promyelocytic leukemia) gene was originally identified in acute PML.^18^ PML and the PML nuclear domain have been regarded as a tumor‐suppressive role in several different types of cancer.^19^ Zhang et al. have found decreased PML protein expression in SCLC. Furthermore, there is evidence that PML was involved in regulation of innate immune response through affecting interferon and targeting cytokines secretion, such as pro‐inflammatory cytokines IL‐1β and IL‐6,^20^, ^21^ thus OS difference between high‐ and low‐risk patients might be partly ascribed to the regulatory role of PML on innate immune signaling in these groups. Besides, the roles of seven genes (*NR1D2*, *TANK*, *LRSAM1*, *PLXNA1*, *INHBE*, *HDGF*, and *ARAF*) in SCLC have not been reported, however those genes have been reported to play a vital role in other type cancer.^22^

Furthermore, we attempted to investigate the potential molecular background of the prognostic immune‐related signature. Go and KEGG pathways were further analyzed and proved the robust connection of the signature with growth factor activity and immune‐regulatory TGF‐β signaling pathway. Unlike NSCLC, SCLC had different expression levels of TGF‐β and its receptors. Autocrine and paracrine growth inhibition by TGF‐β has been found in SCLC because of the inhibitory synthesis of TGF‐β isoforms and TGF‐β II.^23^ In addition, SCLC cell lines suppressed IL‐2‐dependent T cell growth via secreting active TGF‐β1.^24^ A specific anti‐TGF‐β1 antibody or a recently developed novel bifunctional anti‐PD‐L1/TGF‐β checkpoint inhibitor, the fusion protein M7824, decreased tumor burden and increased survival in mice through promoting CD8^+^ T cell and NK cell activation and blocking the immunosuppressive activity induced by the SCLC cells.^24^, ^25^ Therefore, blockade of TGF‐β pathway represents a novel therapeutic strategy for SCLC in terms of combination immunotherapy.

CD56^dim^ NK cells possess a strong cytolytic capacity, but with low levels of cytokines production.^26^, ^27^ Picard et al. have found that lower rate of the cytotoxic CD56^dim^ CD16^+^ NK cells was observed in NSCLC patients compared with healthy control, indicating CD56^dim^ NK cells play an important role in cancer immunosurveillance.^28^ NanoString transcriptomic analysis of melanomas revealed that there was a trend of increased CD56^dim^ NK cell gene signature expression associated with better clinical outcome.^29^ In the sophisticated, genetically engineered mouse models, Best et al. found that the lack of NK cells, but not CD8^+^ T cells, substantially promote metastatic dissemination of SCLC tumor cells in vivo,^30^ indicating that NK cells play a vital role in the prognosis of SCLC patients. In our study, in total population we observed that the abundance of CD56^dim^ but not CD56^bright^ NK cells is positively associated with the increased survival of SCLC patients, and the abundance of pDC is inversely associated with the increased survival. Therefore, CD56 ^dim^ and CD56 ^bright^ NK cells might differentially affect the prognosis of SCLC patients. It has been reported that CD56^bright^ NK cells inversely correlate with the survival of melanoma patients, also IFN‐γ production from CD56^bright^ NK cells correlated inversely with the OS of patients,^31^ however, the comprehensive role of the subpopulation of NK cells in SCLC has not yet been clarified.

It has been widely observed that tumor‐associated pDCs are associated with an increase in Tregs and the decrease in OS in gliomas,^32^ ovarian,^33^ and breast cancer,^34^ and lung cancer. Sorrentino et al. have found that depletion of pDCs with a specific antibody (m927) in a mouse model of Lewis lung carcinoma cell‐induced lung cancer reversed the immune‐suppressive microenvironment, including decreased tumor burden, activation of mDC and CD8^+^ T cells, and Th1‐ and Th17‐like cytokine production.^35^ Additionally, Munn et al. have found that a subset of pDCs in mouse tumor‐draining lymph nodes that constitutively expressed immunosuppressive indoleamine 2,3‐dioxygenase suppressed T‐cell responses and induced T anergy.^36^ Thus, these results indicate an unappreciated level of heterogeneity of SCLC immunophenotype associated with diverse clinical outcome.

We also analyzed the immune cell infiltration profile for both low‐risk and high‐risk patients. We failed to observe a significant difference in CD56^dim^ NK cells and pDC subset between low‐risk and high‐risk groups. However, patients with high risk had more CD8^+^ T cells, helper T cells, mast cells, and Tfh cells but less Treg cells compared to those with low risk. Although some studies showed that tumor‐associated CD45‐positive cells,^37^, ^38^ tumor‐infiltrating lymphocytes,^37^ and CD8^+^ T cells^39^ in SCLC specimens were a good clinical marker to identify patients with favorable prognosis, but there was no significant association between CD45‐positive cell counts and advanced disease stage.^40^ In addition, high‐risk patients had a high level of mast cells that have been found to relate to unfavorable survival.^41^ Interestingly, our results demonstrated that patients with low risk had more CD56^bright^ cell subset that were responsible for large amounts of pro‐inflammatory cytokines production but not cytotoxic ability than patients with high risk. Thus, at least the present cohort reflected a different immunological microenvironment in SCLC patients with diverse prognosis.

Taken together, unlike NSCLC and other solid tumors, the immune microenvironment of SCLC is characterized as few tumor‐infiltrating lymphocytes and low PD‐L1 expression. Nevertheless, immunotherapy with immune‐checkpoint inhibitors still holds promise for SCLC patients independent of PD‐L1 expression status.^42^ Therefore, it is essential to characterize SCLC patients who have a poor prognosis or benefits from immune checkpoint blockade, and the future research focusing on the identification of predictive biomarkers of prognosis and efficacy of immunotherapy and the characteristics of the SCLC immune microenvironment is urgently needed.^42^ In this study, we constructed the 10‐gene signature which successfully predict patients’ prognosis and validated its accuracy in SCLC.

Our research has certain limitations. First, this study was based on bioinformatics analyses from one public database with a limited number of patients, which indeed weaken the strength of our findings. Second, of 10 immune‐related genes in our study, the role of three genes in SCLC has been investigated; however, the roles of other seven genes in SCLC have not been identified. Third, it is hard to validate the predictive value of our model in immunotherapy for SCLC patients as a lack of treatment‐related information. In future, the expression and function of immune‐related genes in SCLC tumor cells or infiltrated immune cells within tumor should be elucidated. The predictive value of this immune‐related signature should be further validated using different or real‐word SCLC cohorts with larger patient size, especially with detailed information on immunotherapy for SCLC patients. Flow cytometry and real‐time quantitative PCR as alternative tools should be used to verify our findings.

## ETHICS APPROVAL AND CONSENT TO PARTICIPATE

Not applicable.

## CONFLICT OF INTEREST

Author Jian Yi, Hui Yu, and Tiantian Gu were employed by the company Yuce Biotechnology Co., Ltd. (Shenzhen, China). The remaining authors declare that the research was conducted in the absence of any commercial or financial relationships that could be construed as a potential conflict of interest.

## Supporting information

Supplementary Material

## Data Availability

The datasets used and/or analyzed during the current study are available from the corresponding author upon reasonable request.
